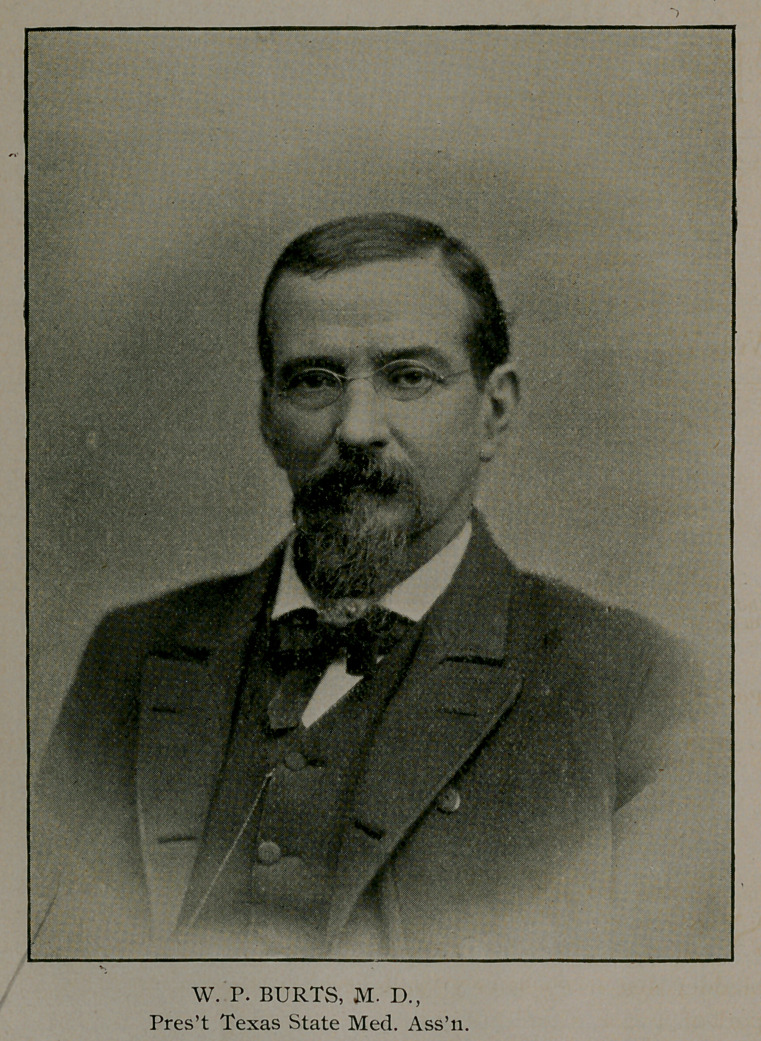# W. P. Burts, M. D., President Texas State Medical Association

**Published:** 1890-06

**Authors:** 


### W. P. BURTS, M. D., PRESIDENT TEXAS STATE MEDICAL ASSOCIATION.

---

Dr. Burts, the President of the Texas State Medical Association, whose portrait graces this edition of the JOURNAL, was born at Green Meadow, Washington county, Tennessee. He was born on his father's farm—"Green Meadow," December 7, 1827. He had only the advantages afforded by the schools of the country at that early day, until he was "a good sized boy," when he was sent to Martin Academy at Jonesboro. Here he studied Latin, Greek, French and Mathematics; later he entered Washington College, and afterwards he spent three years at Tuscumbia College, Tenn. When in his senior year at this college he was seized with a desire to go to California, and quit college for that purpose, but his parents offered so much opposition that the intention was reluctantly abandoned, and the study of medicine was begun.

After a course of preparatory reading he matriculated at Geneva Medical College, New York, and graduated there in 1852. He begun the practice of medicine at Tazewell, Virginia, the same year, and continued there until 1858, when he immigrated to Texas, settling in the then small village of Fort Worth. Here he has resided and practiced medicine to date.

When in 1882 the Texas State Medical Association met in Fort Worth, Dr. Burts was elected First Vice-President. He is one of the oldest members and has always taken an active interest in its affairs. As Chairman of the Committee of Arrangements for the last meeting he gave great satisfaction. At the Houston meeting, April, 1885, he was Chairman of the Section on Surgery, and made an elaborate and very able address, reviewing the progress of the science for the last two decades. This is published in the Transactions of that session. He has served as President of the Fort Worth and Tarrant County Medical and Surgical Society, and also of the North Texas (District) Medical Association. He is an old member of the American Medical Association, and has several times represented the Texas State Medical Association as a delegate to that body. As a token of the esteem in which he is held by his confreres—at the last meeting he was, by acclamation, elected to the highest office within their gift.

Dr. Burts is universally esteemed as an able physician, and his practice extends over a large area of North Texas, he being frequently called in consultation hundreds of miles from Fort Worth. As a citizen and a physician he is universally respected, and the Texas State Medical Association honors itself in his election as President.

## Figures and Tables

**Figure f1:**